# Delineation of cystinuria in Saudi Arabia: A case series

**DOI:** 10.1186/s12882-017-0469-x

**Published:** 2017-02-06

**Authors:** Abdulrahman Obaid, Marwan Nashabat, Khalid Al Fakeeh, Abdullah T. Al Qahtani, Majid Alfadhel

**Affiliations:** 1 King Abdullah International Medical Research Centre, King Saud bin Abdulaziz University for Health Sciences, Genetic Division, Department of Pediatrics, King Abdulaziz Medical City, Ministry of National Guard-Health Affairs (NGHA), PO Box 22490, Riyadh, 11426 Saudi Arabia; 2 King Abdullah International Medical Research Centre, King Saud bin Abdulaziz University for Health Sciences, Nephrology Division, Department of Pediatrics, King Abdulaziz Medical City, Ministry of National Guard-Health Affairs (NGHA), PO Box 22490, Riyadh, 11426 Saudi Arabia

**Keywords:** Cystinuria, Renal calculi, *SLC3A1*, *SLC7A9*

## Abstract

**Background:**

Cystinuria is an inherited metabolic disease that is caused by defects in two genes, *SLC3A1* and *SLC7A9,* which result in a renal reabsorptive defect of cystine and other dibasic amino acids, including ornithine, arginine, and lysine. Patients usually present with recurrent renal calculi and may develop renal impairment. Medical management includes high fluid intake and chelating agents. To the best of our knowledge, this is the first study describing cystinuria in Saudi Arabia.

**Methods:**

A retrospective chart review for cystinuria patients from the genetic and nephrology divisions between 2010 to 2015. All patients were investigated, diagnosed and treated at King Abdulaziz Medical City in Saudi Arabia.

**Results:**

Eight patients were identified from five unrelated families. The age of onset ranged from birth to 14 years. The female to male ratio was 1.7:1. Two new variants in the *SLC3A1* and *SLC9A7* genes were discovered. All of the detected mutations were missense variants in three different exons, such as c.1711 T > A (p.Cys571Ser) (exon 10), c.1166C > T p.Thr389Met (exon 11) and c.1400 T > A p.Met467Lys (exon 8). Additionally, 37.5% of our patients developed arterial hypertension and 25% had urinary tract infection, but none had renal impairment. No significant clinical differences were detected in this study between type A (*SLC3A1* variants) and type B cystinuria (*SLC7A9* variant). Two cases were diagnosed based on clinical information, biochemical testing and a positive family history as all of the molecular testing for cystinuria was negative.

**Conclusion:**

Cystinuria has wide genetic heterogeneity with a poor genotype/phenotype correlation. Negative molecular investigations should not rule out the disease if clinical and biochemical investigations support the diagnosis. A larger data registry is essential to better describe the cystinuria genotype/phenotype in Saudi Arabia.

## Background

Cystinuria continues to be one of the most challenging stone diseases [1], which has a recurrence rate up to 60%. It is associated with progressive renal impairment [2]. Cystinuria is one of four genetic metabolic diseases discovered by Carrod in 1908 [3]. A recent study published from our center predicted the incidence of cystinuria to be 4.5/100,000 live births [4].

Thus far, cystinuria is thought to be caused by defects in two genes, *SLC3A1* and *SLC7A9. SLC3A1* (MIM #104614) is located on chromosome 2 (2P16.3) and encodes the heavy subunit of the renal amino acid transporter (rBAT) needed to localize the transporter to the plasma membrane. The *SLC7A9* gene (CSNU3, MIM #604144) is located on chromosome 19 (19q13.1) and encodes the light subunit of the renal amino acid transporter (b0,+AT), which compromises the catalytic, transporting component. The Rbat/B0,+AT is linked by a disulfide bridge [5–7]. The mode of transmission seems to be autosomal recessive or autosomal dominant with incomplete penetrance [8, 9].

Amino acid transport defects cause hyper-excretion and insolubility of cystine and other dibasic amino acids as well as precipitation of cystine at the physiological pH of urine, which results in recurrent cystine renal calculi, obstructive uropathy, hypertension, infection and rarely renal failure [10]. More than 50% of cystinuric patients develop cystine urolithiasis, and 75% of them have bilateral stones. Recurrent stone formation requires repeated urological interventions [11, 12].

The diagnosis of cystinuria is achieved by functional, biochemical or molecular investigations. Functionally, patients excreting cystine more than 120 mmol/mol creatinine in 24 h urine are defined as having cystinuria [3]. Biochemically, cystinuria is divided into two subtypes based on the amount of cystine excreted in the urine. For type 1 cystinuria, which has an autosomal recessive mode of transmission, heterozygotes have normal amino acid excretion. On the other hand, for non-type 1 cystinuria, heterozygotes have cystine hyper-excretion, indicating an autosomal dominant mode of transmission with incomplete penetrance [9]. Molecularly, mutations in the *SLC3A1* gene cause type A cystinuria, while type B is due to *SLC7A9* gene mutations. A possible third type, AB, has mutations in both genes [8]. Although it was proposed that *SLC3A1* mutations (type A) cause type 1 cystinuria and *SLC7A9* mutations (type B) cause non-type 1 cystinuria, an increasing number of *SLC3A1* variants were discovered to cause cystine and other dibasic hyper-aminoaciduria in carriers in addition to several point mutations in *SLC7A9*, resulting in silent heterozygotes [10].

The treatment success in cystinuria patients depends on full compliance with medical therapy, including urine alkalization with potassium citrate and dietary sodium restriction. High fluid intake has been shown to decrease urinary stone formation in up to 67% of patients with cystinuria, achieving a urine specific gravity of 1.010. The most effective chelating agent for cystinuria is oral thiol (β,β-dimethylcystine), which forms mixed disulfides with cystine, dissolving and reducing new cystine stone formation [13].

In this study, we described the clinical phenotype and genotype of eight Saudi patients with cystinuria. To the best of our knowledge, this is the first study in the literature describing the clinical, biochemical and molecular characteristics of cystinuria in Saudi Arabia.

## Methods

A retrospective chart review of eight Saudi cystinuric patients from five unrelated families between 2010 to 2015. All patients were diagnosed and treated at King Abdulaziz Medical City in Saudi Arabia under the care of a multidisciplinary team, including nephrologists and geneticists.

The diagnosis was clinically established based on recurrent cystine renal calculi formation that started in childhood. Other biochemical, radiological and molecular investigations were requested to confirm the diagnosis. Investigations that were performed include abdominal X-Ray, ultrasound, abdominal CT, stone analysis, urinary amino acid study and molecular genetic testing. Amino acids in urine were measured using early morning fresh urine samples and 24-h urine. DNA sequencing analysis, coding sequences of the *SLC3A1* and *SLC7A9* genes and mutational analysis were performed at referenced commercial labs. The *SLC3A1*(NM_000341) and *SLC7A9* (NM_014270) genes were analyzed by PCR and sequencing of both DNA strands of the coding introns and highly conserved exon-intron splice junctions.

## Results

### Clinical data (Table 1)

**Table 1:** Clinical data for the cases

Case	Current Age (Years “y”)	Gender	Affected Relatives	Age at Diagnosis	UTI	HTN	Renal Stones	Medical Treatment	Urology Intervention	Note
Case 1	10 y	female	Father, Two sisters, brother	Birth	Yes	No	Bilateral	Tiopronin and potassium citrate	Percutaneous Nephrolithotomy Shock wave lithotripsy	Short Factor V deficiency in affected family members
Case 2	21 y	male	sister	Birth	No	No	Bilateral	Potassium citrate Tiopronin	Right nephrectomy Percutaneous Nephrolithotomy	
Case 3	23 y	Female	brother	12 years	No	No	Right side	Potassium citrate Tiopronin	ESWL	
Case 4	10 y	Female	none	3 years	Yes	Yes	Bilateral	Tiopronin potassium citrate & Amlodipine Lisinopril	Percutaneous Nephrolithotomy	
Case 5	29 y	Female	Paternal Cousin	18 months	No	Yes	Bilateral	Tiopronin Potassium Citrate Enalapril	Percutaneous Nephrolithotomy	
Case 6	15 y	Male	Grandmother	10 years	No	Yes	Bilateral	Penicillamine and potassium citrate	Percutaneous Nephrolithotomy	Vitiligo
Case 7	11 y	Female	Grandmother Brother, sister	10 years	no	No	Unilateral	Penicillamine and potassium citrate	Waiting for Percutaneous Nephrolithotomy	
Case 8	16 y	Male	Grandma Brother and sister	14 years	no	no	unilateral	Penicillamine and potassium citrate	none	Right-sided undescended testis

*UTI* Urinary tract infection and *HTN* Hypertension

All patients were products of consanguineous parents. They presented with renal stone related signs and symptoms; five patients presented with bilateral renal calculi and three had unilateral stones. The ages of onset were variable, ranging from birth to 14 years (mean 6.3 years). The current age of the oldest patient is 24 years. The female to male ratio was 1.7:1. A positive family history was documented in seven cases. Three cases developed hypertension and two cases had recurrent urinary tract infections (UTI).

All parents were asymptomatic. Renal ultrasound and urine amino acids were done for the parents and the results were negative.

### Biochemical and molecular features (Table 2)

**Table 2:** Biochemical and molecular features

	Biochemical				Molecular		
	Amino acids^a^				SLC3A1	Exon	SLC9A7
Cases	Cys.	Arg.	Orn.	Lys.			
Case 1	160	790	177	1179			
Case 2	98	337	163	267	c.1711 T > A p.571Cys571Ser	10	
Case 3	310	3885	508	772	c.1711 T > A p.571Cys571Ser	10	
Case 4	211	558	350	718			
Case 5	211	224	97	357		11	c.1166 C > T p.Thr389Met
Case 6	177	557	235	594	c.1400 T > A p.Met467Lys	8	
Case 7	112	244	108	197	c.1400 T > A p.Met467Lys	8	
Case 8	93	177	83	158	c.1400 T > A p.Met467Lys	8	

^a^Cut off: cystine: 4–12 μmol/mmol crea.; ornithine: 0–6 μmol/mmol crea.; arginine 0–6 μmol/mmol crea.; and lysine: 10–56 μmol/mmol crea

All cases had high urinary dibasic amino acids levels at diagnosis with variable severity. Stone analysis showed yellow–white formation with waxy lusters and cystine. The renal profile remained within normal limits for all patients.

Molecular study showed previously unreported missense variant in exon 10 of *SLC3A1* gene (c.1711 T > A p.Cys571Ser) in two cases (Patients 2 and 3). This variant is located in a highly conserved nucleotide and amino acid position with moderate physiochemical differences between the amino acid cysteine and serine. Software analyses by Polyphen-2 and Mutation Taster predicted that the mutation is probably damaging. Another new variant in exon 11 of the *SLC9A7* gene (c.1166 C > T p.Thr389Met) was found in one patient (Patient 5). This variant was also located in a highly conserved nucleotide and moderately conserved amino acid position, and there were moderate physiochemical differences between the amino acids threonine and methionine. Software analyses by Polyphen-2 and SIFT predict this variant is probably damaging. A previously reported mutation in exon 8 of the *SLC3A1* gene (c.1400 T > A p.Met467Lys), described as disease-causing by Calonge in 1994 (PMID 8054986), was observed in three patients.

### Imaging features (Table 3)

**Table 3:** Imaging features

Case	DMSA	Renal CT	Renal US
Case 1	Right kidney: Relatively Reduced volume, Split renal function 61% Left kidney. Cortical scarring at the upper pole & large photopenic area in the lower pole with Split renal function 39%	Right kidney: large staghorn branching stone on the renal pelvis Left kidney: left renal lower pole stone	Right kidney: grade 3 hydronephrosis Left kidney: grade 2 hydronephrosis Bilateral multiple renal stones.
Case 2	Right kidney small with decrease uptake and small scar Left good kidney uptake with split renal function 92%	Right kidney: Atrophic small right kidney with at least two calculi at its lower pole Left kidney: A tiny calculus at the left vesicoureteric junction.	Right kidney: Small size, lower pole mild to moderate hydronephrosis. Left kidney: non-obstructing calculus noted at the lower pole with mild hydronephrosis.
Case 3		Right kidney: obstructive right periureteric junction stone with moderate hydronephrosis. Left kidney: normal	Right kidney: calculi in the pelvis with moderate hydronephrosis.
Case 4		Right kidney: large stone in the renal pelvis. Small calculus in the lower pole of an atrophic, small kidney. Left kidney: large calculus in the left renal pelvis. Severe hydronephrosis and thinning of the cortex. Multiple non-obstructing calculi seen in the lower pole.	Right kidney: grade 2 hydronephrosis, multiple non-obstructing stones. Left kidney: grade 4 hydronephrosis with thin renal parenchyma and an obstructive stone
Case 5		Bilateral renal stones	
Case 6	Normal right kidney according to DMSA scintigraphy, with reduced left kidney function, hydronephrotic changes, and a scar.		Right kidney: normal Left kidney: multiple stones and grade 1 hydronephrosis.
Case 7		Left non-obstructive renal stones.	
Case 8			Right Kidney: Calculi in the pelvis Left Kidney: Normal

All discovered stones were radio-opaque. According to abdominal X-ray, renal ultrasound, and abdominal CT scan studies, we found six cases with multiple bilateral cystine calculi of variable sizes; two of them were staghorn and two cases had unilateral, multiple stones. DMSA scanning showed a unilateral decrease in renal function that had mild severity in two cases and an atrophic small right kidney in another two cases.

### Course of the disease

Currently, all patients are in stable condition with variable responses to treatment; five patients were on regular treatment with Tiopronin and potassium citrate and three patients were on penicillamine and potassium citrate. Only one male patient underwent right nephrectomy. Six patients were surgically treated by percutaneous nephrolithotomy.

## Discussion

Cystinuria is a rare genetic disease caused by a proximal renal reabsorptive defect of filtered cystine leading to recurrent stone formation [14]. In our study, the age of onset was variable, ranging from birth to 14 years (mean 6.3 years), which is partially consistent with the expected age of the onset of stone formation in cystinuria in the first decade of life and continues lifelong, peaking during the third decade [8, 15]. In a study on Sudanese patients, the mean age at presentation with cystine stones was 31.9 months (range 3–125 months) [16].

In cystinuria, the male to female ratio used to be nearly equal [9]. On the other hand, in our study, females were affected nearly twice as much as males (ratio1.7: 1). Other studies in the Middle East reported a reverse finding with a male to female ratio of 2.3:1 [16]. Two of our patients developed staghorn calculi, supporting the expected natural course of the disease, and they had recurrent and multiple bilateral renal stones [15]. The structure and composition of the stones of our patients mimic the typical composition of cystine stones [16].

Furthermore, 34% of patients with cystine calculi might present with signs of UTI and hypertension; there can be renal failure in up to 17% [6, 7]. In our study, two patients (25%) presented with renal calculi and UTI. Hypertension developed in three cases (37.5%), but there was no renal impairment, which might be due to early detection and proper management.

Although the most frequent mutation in the Mediterranean region in the *SLC3A1* gene is M467T [17], three of our patients (37.5%) from a single family were homozygous for the less common M467L mutation, which was previously reported by Calonge, M. et al. (1995) as compound heterozygous. Different regions of the world show characteristic mutation frequencies; for example, p.T216M in *SLC3A1* and p.G105R in *SLC7A9* are common in Greece [18]. In Portuguese, deletion and duplication (accounts 33.3%), especially E5_E9 Dup, are the most common, while c.1400 T > C p.M467T accounted for 11.1% of mutated alleles. This reflects the importance of screening for large deletion/duplication mutations in cystinuria patients. Furthermore, even if patients were negative, the negative finding does not rule out the disease [10]. Two of our patients (25%) did not show any pathogenic mutations in the target genes; moreover, their deletion/duplication study was also negative. As a result, the patients were diagnosed based on clinical and biochemical results.

In our study, all detected variants were caused by point mutations of the missense type. This is in contrast with previously reported mutations that are nonsense, splicing, small rearrangement and gross rearrangement mutations (insertion and deletion) [10]. Thus far, more than 160 mutations have been discovered in the *SLC3A1* gene and more than 116 mutations have been discovered in the *SLC7A9* gene. [1].

We identified two novel mutations in the *SLC3A1* and *SLC7A9* genes. Both variants where located in highly conserved positions, and they are predicted to be damaging according to software analyses. Additionally, they segregated well in the families, which supports the causality for the patient’s phenotype. These newly described variants support and expand on the allelic heterogeneity and complexity of genes variants previously described in cystinuria [19]. The phenotypes of the newly discovered mutations mimic the classical cystinuria phenotype.

Although the missense variant, G105R, has been reported as one of the most frequent mutations in the *SLC7A9* gene in cystinuria patients, mainly in Europe [9], it was not found in any of our patients.

In our study, the long-term complications of cystinuria were less than those mentioned in the literature. This may be explained by the early detection and aggressive management of disease. Accordingly, we recommend early screening and diagnosis for other children from affected families.

## Conclusion

The newly described mutations result in a classical cystinuria picture. The most frequent mutation discovered in our study was in the *SLC3A1* gene, M467L. Negative molecular testing does not rule out the disease if patients are symptomatic. Cystinuria should be considered in each child who has a renal stone and UTI with or without positive family history. Some patients might develop hypertension and UTI, but they do not necessarily have end-stage renal disease especially if diagnosed early and managed properly. No significant clinical differences were detected in this study between types A and B cystinuria. Screening for children in affected families is recommended. A larger data registry is essential for better describing the cystinuria genotype/phenotype in Saudi Arabia.

## Abbreviations

CT: Computed topography
DMSA: Dimercaptosuccinic acid
PCR: Polymerase chain reaction
UTI: Urinary tract infection

## References

[1] Andreassen KH, Pedersen KV, Osther SS, Jung HU, Lildal SK, Osther PJ (2016). How should patients with cystine stone disease be evaluated and treated in the twenty-first century?. Urolithiasis.

[2] Knoll T, Zollner A, Wendt-Nordahl G, Michel MS, Alken P (2005). Cystinuria in childhood and adolescence: recommendations for diagnosis, treatment, and follow-up. Pediatr Nephrol.

[3] Gitomer WL, Reed BY, Ruml LA, Skhaee K, Pak CYC (1998). Mutations in the genomic deoxyribonucleic acid for SLC3A1 in patients with cystinuria. J Clin Endocrinol Metab.

[4] Alfadhel M, Benmeakel M, Hossain MA, Al Mutairi F, Al Othaim A, Alfares AA, Al Balwi M, Alzaben A, Eyaid W (2016). Thirteen year retrospective review of the spectrum of inborn errors of metabolism presenting in a tertiary center in Saudi Arabia. Orphanet J Rare Dis.

[5] Eggermann T, Venghaus A, Zerres K: Cystinuria: an inborn cause of urolithiasis. *Orphanet Journal of Rare Diseases* 2012, 7(19). 10.1186/1750-1172-7-19 PMC3464901 22480232

[6] Feliubadaló L, Font M, Purroy J, Rousaud F, Estivill X, Nunes V, Golomb E, Centola M, Aksentijevich I, Kreiss Y (1999). Non-type I cystinuria caused by mutations in SLC7A9, encoding a subunit (bo,+AT) of rBAT. Nat Genet.

[7] Calonge MJ, Gasparini P, Chillarón J, Chillón M, Gallucci M, Rousaud F, Zelante L, Testar X, Dallapiccola B, Silverio FD (1994). Cystinuria caused by mutations in rBAT, a gene involved in the transport of cystine. Nat Genet.

[8] Strologo LD (2002). Comparison between SLC3A1 and slc7a9 cystinuria patients and carriers: a need for a new classification. J Am Soc Nephrol.

[9] Koulivand L, Mohammadi M, Ezatpour B, Salehi R, Markazi S, Dashti S, Kheirollahi M (2015). Mutation analysis of SLC3A1 and SLC7A9 genes in patients with cystinuria. Urolithiasis.

[10] Barbosa M, Lopes A, Mota C, Martins E, Oliveira J, Alves S, De Bonis P, Mota Mdo C, Dias C, Rodrigues-Santos P (2012). Clinical, biochemical and molecular characterization of cystinuria in a cohort of 12 patients. Clin Genet.

[11] Pras E (2000). Cystinuria at the turn of the millennium: clinical aspects and new molecular developments. Mol Urol.

[12] Pierides AM, Deltas CC (1997). Clinical aspects of cystinuria. Hereditary Kidney Dis.

[13] Dahlberg P, Berg V, Kurtz S, Wilson D, Smith L (1977). Clinical features and management of cystinuria. Mayo Clin Proc.

[14] Al-Marhoon MS, Bayoumi R, Al-Farsi Y, Al-Hinai A, Al-Maskary S, Venkiteswaran K, Al-Busaidi Q, Mathew J, Rhman K, Sharif O (2015). Urinary stone composition in Oman: with high incidence of cystinuria. Urolithiasis.

[15] Al-Hermi B, Abbas B (2003). Cystinuria in Arab Countries. Saudi J Kidney Dis Transplant.

[16] Elfadil GA, Ibrahim ME, Elmugadam AA, Ahmed SAM (2014). Urinary cystine calculi and detection of polymorphism in the SLC3A1 gene in Sudanese children. J Taibah Univ Med Sci.

[17] Botzenhart E, Vester U, Schmidt C, Hesse A, Halber M, Wagner C, Lang F, Hoyer P, Zerres K, Eggermann T (2002). Cystinuria in children: distribution and frequencies of mutations in the SLC3A1 and SLC7A9 genes. Kidney Int.

[18] Chatzikyriakidou A, Louizou E, Dedousis GV, Bisceglia L, Michelakakis H, Georgiou I (2008). An overview of SLC3A1 and SLC7A9 mutations in Greek cystinuria patients. Mol Genet Metab.

[19] Brons AK, Henthorn PS, Raj K, Fitzgerald CA, Liu J, Sewell AC, Giger U (2013). SLC3A1 and SLC7A9 mutations in autosomal recessive or dominant canine cystinuria: a new classification system. J Vet Intern Med.

